# Filipino Multicomponent Intervention to Maintain Cognitive Performance in High-Risk Population (FINOMAIN): Study Protocol for a Cluster Randomized Controlled Trial

**DOI:** 10.3389/fneur.2021.685721

**Published:** 2021-09-07

**Authors:** Jacqueline Dominguez, Ma. Fe de Guzman, S. H. Annabel Chen, Mary Sano, Gunhild Waldemar, Thien Kieu Thi Phung

**Affiliations:** ^1^St. Luke's Medical Center, Institute for Neurosciences, Quezon City, Philippines; ^2^Institute for Dementia Care Asia, Quezon City, Philippines; ^3^Center of Research and Development in Learning, Psychology, School of Social Sciences, Lee Kong Chian School of Medicine, Nanyang Technological University, Singapore, Singapore; ^4^Department of Psychiatry, Mt. Sinai Alzheimer's Disease Research Center, Icahn School of Medicine, New York, NY, United States; ^5^Danish Dementia Research Center, Rigohospitalet, University of Copenhagen, Copenhagen, Denmark; ^6^Department of Clinical Medicine, Faculty of Health and Medical Sciences, University of Copenhagen, Copenhagen, Denmark

**Keywords:** mild cognitive impairment, cluster-randomized trial, multicomponent, dance, Philippines

## Abstract

**Background:** More than half of the people with dementia live in lower-middle income countries (LMIC), yet we lack research and evidence-based knowledge to guide health promotion and prevention strategies for cognitive decline. In the Philippines, the prevalence of mild cognitive impairment (MCI) and cardiovascular risk factors among older persons are high, making this population at high risk for developing dementia. This protocol describes a cluster randomized controlled trial that aims to investigate the efficacy of a multicomponent intervention to maintain cognitive performance among high-risk population.

**Methods:** This is a cluster-randomized, two-arm, single-blind trial of a multicomponent intervention that combines dance called INDAK (Improving Neurocognition through Dance and Kinesthetics), nutrition counseling, and vascular risk management. The intervention arm will receive 12 months (1-h, twice per week) of INDAK and every 3 months of nutrition counseling and intensive vascular risk management and monitoring. The control group will receive the usual vascular care advice and referral. A total of 605 (20–25 clusters per arm) community-dwelling Filipino older adults aged ≥ 60 years old with MCI will participate in the study and will be assessed at baseline, 6th- and 12th-month follow-up. The primary outcome is cognitive performance assessed by the Alzheimer's Disease Assessment Scale—Cognitive (ADAS-Cog), Mnemonic Similarity Tasks (MST), and executive function composite (EFC). Secondary outcomes are functional connectivity assessed through brain imaging, and measures of behavioral, functional level, and quality of life.

**Discussion:** The study aims to provide scientific evidence on a public health intervention that is contextualized in a community setting to reduce dementia risk among older adults with MCI. This model can be an ecological, low-cost, and effective program, thereby conducive to widespread implementation in the Philippines as well as in other low-resource settings with similar public health challenges. The pilot phase was underway with eight villages (clusters), but temporarily interrupted by the pandemic. The full study is anticipated to start after community restrictions are eased.

## Introduction

The Filipino aging population comprised about 7% of the country's total population in 2015 and is projected to increase by 11% in 2025 and 16% in 2045 (1). Like many other lower middle-income countries (LMIC), the Philippines is not only experiencing demographic aging but also undergoing epidemiological transition due to increased unhealthy diets, sedentary lifestyles, and poor healthcare leading to dramatic increase in dementia epidemic and its associated risks (2–4). In a population-based cohort study of Filipino older adults, aged ≥ 60 years, we found a dementia prevalence of 10.6% (5), much higher than the 7% estimate for Southeast Asia (2), and 23.2% had mild cognitive impairment (MCI). Almost half (48.1%) of those with baseline MCI status, from the same cohort, developed incident dementia after 3–4 years of follow-up (6) indicating MCI as a potentially progressive condition that makes older individuals at high risk for developing dementia (7). Progression from MCI to dementia occurs at a higher rate with an estimated conversion rate of 10–15% per year (7, 8). The odds of progressing to dementia is increased by 3.07 times among those with MCI (6) and further increased by 1.53–2.95 times higher if identified with comorbid conditions such as cardiovascular risks and metabolic syndrome (9). This condition is prevalent in Filipino older adults, with around 82.0% of men and 70.4% of women having at least one cardiovascular risk factor (hypertension, diabetes, dyslipidemia, and smoking) (5). Mixed pathologies and comorbidities are common in the MCI population; therefore, multicomponent treatment strategy is a reasonable approach (7, 10).

Addressing several modifiable risks factors potentially accounts for 40% case preventions or delays in dementia incidence, acknowledging that cognitive decline is a multifactorial phenomenon (11). Complex interventions that address multiple risk factors are limited, but demonstrated promising results (12, 13). One randomized controlled trial (RCT) assessing the efficacy of multicomponent intervention combining exercise, cognitive training, and management of cardiovascular risk factors, the Finnish Geriatric Intervention Study to Prevent Cognitive Impairment and Disability (FINGER), demonstrated cognitive benefits at a 2-year interim endpoint (12). The study population were older individuals with high vascular risk scores and cognitive performance slightly lower than population norms (14). Another RCT of a 6-year multidomain vascular care intervention, although it did not reduce the incidence of all-cause dementia, showed potential benefits particularly among participants with high adherence rate and baseline untreated hypertension (13). Two significant insights can be drawn from these studies (12, 13): first is selection of high-risk population who could benefit more from the intervention, and second, combination of physical activity and vascular risk management can provide more cognitive benefits than interventions targeting vascular risks alone, hence, the rationale for designing a multicomponent intervention combining physical activity such as dance (INDAK—Improving Neurocognition through Dance and Kinesthetics) (15), with intensive vascular risk management and monitoring of adherence, and nutrition counseling.

Physical activity has been shown to improve cardiovascular fitness and cognitive function across various domains (16). While dancing seems primarily a physical activity, it offers a complex and enriched environment for older adults (17) by providing increased multisensory and motor stimulations, challenging cognitive tasks, and increased social interaction (15, 17). Promisingly, a well-designed RCT demonstrated through structural magnetic resonance imaging (MRI) that dancing has superior positive long-term effects on neuroplasticity compared with repetitive or routine exercise. This study was conducted among cognitively healthy older adults (18–20); thus, the benefits of dance on brain and cognition for older adults with MCI remains to be studied (21). Dance is a mind and body activity, which is demonstrated to improve global cognitive and executive function (21, 22), as well as improve balance (18, 20), the major control center for which is the cerebellum. Interestingly, a ground-breaking study from Chen and Desmond (23) demonstrated by using functional MRI (fMRI) that cerebellar integrity is associated with cognitive performance highlighting the critical role of cerebellum in non-motor processes expanding the study of neuroplasticity in this area of the brain when studying dance and cognition. It has positive benefits on gross-motor coordination and balance, which can facilitate and maintain the cerebro-cerebellar functional connectivity of the brain. Specifically, Chen and Desmond (23) examined the higher cognitive function of verbal working memory and identified specific regions [inferior frontal gyrus (IFG), the inferior parietal lobule (IPL), and the right superior and inferior cerebellum] to be functionally connected in this network. For visual working memory, Ng et al. (24) found the bilateral dorsal lateral prefrontal cortex (DLPFC), bilateral IPL, and bilateral superior and inferior cerebellum (more so on the left) to be activated. This improved connectivity is hypothesized to improve cerebellar and hippocampal function particularly on working memory, which is a component of executive functions (EFs). We define the core EFs to constitute inhibition, interference control, working memory, and cognitive flexibility (25). A brief battery for executive function composite (EFC) scales and the Mnemonic Similarity Task (MST) (26, 27) will be used to evaluate cognitive changes due to dancing. In order to capture the neuroplasticity of the cerebro-cerebellar networks, we will apply a well-validated, robust visual working memory fMRI task (24). As verbal working memory required the reading of alphabets, we decided to use the visual working memory task instead as it is non-language dependent and has been well-validated in previous studies (24, 28). Although a few studies that analyzed the effect of dance using fMRI have demonstrated enhancement of cortical and subcortical functional connectivity (29–31), these studies were mostly cross-sectional in design, involving young participants. Furthermore, benefits of dance extend beyond neurocognitive benefits by improving physical, functional, socioemotional, and quality of life in older adults (22).

In addition, changes in white matter integrity as measured by diffusion tensor imaging (DTI), intrinsic functional connectivity examined with resting-state fMRI, and brain volume changes examined by voxel-based morphometry (VBM) have been found to be enhanced with physical activities such as dancing in older adults (18–20, 30, 31). We will evaluate how these brain changes are associated with the cognitive measures to understand the effect of dancing on other EFs and memory encoding in our MCI sample.

This study aims to evaluate a community-based intervention program focusing on the effect of dance with intensive management of vascular risks to maintain cognitive performance among older persons with MCI. It further attempts to document its potential underlying neural mechanism through structural and functional MRI. We hypothesize that the intervention group will primarily have better cognitive function and potentially improve functional balance, mood, and quality of life than controls. For task-fMRI, we hypothesize that the functional connectivity of the targeted cerebro-cerebellar networks (DLPFC—superior cerebellum and IPL—inferior cerebellum) involved in visual working memory will be enhanced in the intervention group compared with controls. For resting-state fMRI, we will examine changes in the default mode network, executive control network, and salience network in the intervention group compared with the control group. We expect to see enhanced white matter integrity (examined by diffusion MRI) in related networks in the intervention group compared with the control group. We also hypothesize brain volume increases in frontal brain regions in the intervention group relative to the control group.

## Methods and Analysis

### Study Design

FINOMAIN is a community-based, single-blind (outcome assessor), cluster-randomized controlled trial of a multicomponent intervention with two arms: an intervention and a control arm. The intervention arm will receive 12 months (1 h, twice per week) of dance training called INDAK, and intensive vascular risk management, which includes regular visits with the study physician for treatment and monitoring of vascular risks and nutrition counseling. A waitlisted control group will receive usual vascular care that includes general advice on management of risks and referral to the local health clinic. The protocol was prepared based on SPIRIT (Standard Protocol Items: Recommendations for Interventional Trials) guidelines (32), and the multicomponent intervention design followed the Medical Research Guidelines on the evaluation and development of complex interventions (33). A pilot trial will be carried out to assess the acceptability, safety, and feasibility of this multicomponent intervention. Ethics approval has been granted by St. Luke's—Institutional Ethics Review Committee (CT-18146) and was retrospectively registered at Clinicaltrials.gov with identifier NCT04301544 (trial registration data available at: https://clinicaltrials.gov/ct2/show/NCT04301544).

### Study Population

Participants are community-dwelling Filipinos aged 60 years old and above, with MCI.

#### Inclusion Criteria

- MCI evaluation is based on Petersen criteria (34), i.e., cognitive symptoms documented by standardized cognitive test ≥ 1.5 standard deviation (SD) below Filipino norms for Alzheimer Disease Assessment Scale—Cognitive (ADAS – Cog) (35) but do not fulfill DSM-5 criteria for major cognitive disorders (36) and has a Clinical Dementia Rating (CDR) of 0.5 (37).
- No significant functional impairment based on the Lawton's Instrumental Activities of Daily Living (IADL) scale (38) quantified as no more than 10 points (15).
- No significant neuropsychiatric symptoms as detected by the Neuropsychiatric Inventory— Questionnaire (NPI-Q) (39) quantified as no more than six points (15).
- Able to read and write to complete assessment.
- Able to provide written consent and willingness to comply with study visits/testing over the 12-month intervention and assessment period.

#### Exclusion Criteria

- Major sensory problems such as hearing and visual impairment that limits testing or dance participation.
- Any preexisting illness that in the judgment of the study physician precludes engagement in moderate physical activity.
- Intake of any anticholinergic drugs or cognitive-impairing/-enhancing medications.

### Sample Size Determination

Sample size was calculated based on the results of quasi-experimental study for INDAK where we compared the mean difference in ADAS-Cog, the primary outcome, from baseline and after 1-year follow-up between intervention and control groups. In that study, ADAS-Cog decreased by 3.16 (SD = 5.97) in the intervention group and 0.91 (SD = 8.69) in the control group (15), with an α error of 5% and β of 5% (power of 95%), and a one-tailed alternating hypothesis in favor of the intervention group, the sample size calculated is 136 per group or 272 for two groups (intervention and control). To account for clustering, a design effect of two will be adopted. This means that the total sample will be 544, with 272 participants per group. Allowing for 10% dropout, the final total sample size required in this study is 605 participants. This will be divided into 20–25 clusters per treatment arm. One cluster will have 12–15 participants, a range we found to be optimum for the intervention in the previous observational study. For MRI, at least 20% of the sample will undergo MRI, selected randomly from both intervention and control arm. This will be proportionally allocated to both arms. The estimated pilot size is 60 participants or 30 per group, expecting higher dropouts; 20% allowance was added giving a final pilot size of at least 72 or 36 per group. The pilot phase is to assess the acceptability of combined interventions (i.e., INDAK, vascular risk management, and nutrition counseling), determine adherence and dropout rates, as well as identify challenges and feasibility of conducting and advancing our research findings with neuroimaging outcomes.

### Recruitment and Assessment Visits

Based on power calculations specified above, we aim to enroll a total of 605 community-dwelling participants based on specified inclusion and exclusion criteria, in which, at least 72 participants are part of a pilot trial. At least 12–15 participants will be enrolled from each *barangay* (local community) to form one cluster. Pre-feasibility assessment will be conducted to assess if communities have resources to support recruitment and intervention such as availability of community health workers to assist in the study and venue for INDAK intervention. Recruitment will be done through public advertisements, and mobilizing community health workers will be mobilized to invite participants for screening. Schedule of visits, assessments, and interventions are outlined in Table 1.

**Table 1:** Schedule of enrollment, intervention, and data collection.

**Study schedules**	**Pre-intervention**		**Intervention period**			
	**SV1**	**SV2**	**Month 0 (V1)**	**Month 3**	**Month 6 (V2)**	**Month 12 (V3)**
**Enrollment**
Screening assessment	x					
Informed consent	x					
Eligibility screening		x				
Allocation		x				
**Interventions**
INDAK			x	x	x	x
Nutrition counseling			x	x	x	
Vascular risks management			x	x	x	
**Data to be collected**
Sociodemographic data	x					
AD8	x					
MoCA-P	x					
Medical history		x			x	x
Physical and neurologic examination		x				
CDR		x			x	x
ADAS-Cog		x			x	x
Lawton's IADL		x				
NPI-Q		x				
MST			x		x	x
EFC			x		x	x
GDS			x		x	x
EQ-VAS			x		x	x
BBS			x		x	x
Anthropometric data			x		x	x
FBS			x		x	x
Lipid profile			x		x	x
ECG			x		x	x
APOE genotype			x			
MRI			x			x

*SV1 and SV2, screening visits 1–2; V1, month 0 is baseline; V2, assessment visit 2 done after the 6th month; V3, assessment visit 3 done after the 12th month; AD8, Ascertaining Dementia 8-Item Scale (AD8); MoCA-P, Montreal Cognitive Assessment Philippines; CDR, Clinical Dementia Rating Scale; ADAS-Cog, Alzheimer's Disease Assessment Scale—Cognitive; IADL, Lawton's Instrumental Activities of Daily Living; NPI-Q, Neuropsychiatric Inventory—Questionnaire; MST, Mnemonic Similarity Task; EFC, executive function composite; GDS, Geriatric Depression Scale; EQ-VAS, EuroQoL—Visual Analog Scale; BBS, Berg Balance Scale; FBS, fasting blood glucose; ECG, electrocardiogram; APOE, apolipoprotein-ε genotype; MRI, magnetic resonance imaging.*

*Sociodemographic data include gender, age, education, and income; anthropometric data include blood pressure, heart rate, body mass index (BMI), and abdominal circumference*.

#### Screening Visit 1 (SV1)

Delegated psychologists or nurses will obtain written consent from participants and their caregivers/family members prior to any procedures. Trained community health workers will administer the Filipino version of Ascertaining Dementia 8-item scale (AD8-P) (40), and psychologists will administer the Filipino version of the Montreal Cognitive Assessment (MoCA-P) (41). Participants with AD8-P ≥ 2 and/or MoCA-P ≥ 21 will proceed for the second screening visit. The cutoff scores for AD8-P will be used to determine reports of cognitive impairment (Dominguez JC, manuscript submitted), while the cutoff scores for MoCA-P will be used to ensure that participants are not suspected to have dementia (41). Further screening will be done on SV2 to determine if cognitive impairment is sufficient to be diagnosed as MCI.

#### Screening Visit2 (SV2)

This is conducted within a week from SV1 to determine participant eligibility based on inclusion and exclusion criteria. The assessment team is comprised of a neurologist who will do a complete medical history, physical and neurologic examination, and the CDR (37), a nurse who will administer the NPI-Q and IADL to the collateral informant, and a psychologist who will administer ADAS—Cog to the participant to confirm a performance score of ≥1.5 SD below average.

#### Baseline Assessment (V1)

Once deemed eligible by the neurologist, the psychologist performs full neuropsychological test battery, described in detail at a section for outcome measures. For baseline data, nurses will collect information on sociodemographics (age, education, and income), vascular risk factors (hypertension, diabetes, dyslipidemia, cardiovascular diseases, cerebrovascular diseases, smoking, alcohol consumption, and physical inactivity), medical history and examination, medications, and anthropometric data (height, weight, abdominal circumference, blood pressure, pulse rates, etc.). The Berg Balance Scale will be conducted by a physical therapist to assess balance. Baseline laboratory tests include fasting blood sugar (FBS), lipid profile, electrocardiogram (ECG), apolipoprotein-ε (APOE) genotype, and MRI for all participants will be conducted within 7–14 days after V1.

#### Mid-Intervention Assessment Visit (V2, 6th Month)

The psychologist will administer the primary and secondary outcome measures, except the BBS, which will be done by the physical therapist. Laboratory tests will be done on all participants to monitor and guide vascular risk management.

#### Post-Intervention Assessment Visit (V3, 12th Month)

The psychologist will administer the primary and secondary outcome measures, while the physical therapist will conduct the BBS. Laboratory tests will be repeated on all participants to monitor response to vascular risk management. Repeat MRI will only be done at V3. To ensure blinding, participants are asked not to discuss any intervention with the assessor.

### Outcome Measures

#### Primary Outcome Measures

- Alzheimer's Disease Assessment Scale—Cognitive (ADAS-Cog) (42): This test consists of 11 tasks measuring the core cognitive deficits of Alzheimer's disease (AD), such as disturbances of memory, language, praxis, attention, visuospatial function, and other cognitive abilities. The ADAS-Cog was validated for use in the Filipino elderly with age- and education-stratified norms (35, 42). Its psychometric properties have good reliability (e.g., Cronbach's α 0.84; test–retest intraclass correlations/ICC: 0.93) and validity (MMSE-P: −0.88 and CDR: 0.81) (42, 43). Score ranges from 0 to 70 points, with lower scores indicating better cognitive function.
- Mnemonic Similarity Task (MST) (27): This task places strong demands on pattern separation, memory representation, and cognitive domains highly correlated with hippocampal function. It consists of two phases: encoding and test part. The encoding phase presents 128 colored photographs of objects on a computer screen to be classified by participants as either indoor or outdoor objects. The test phase consists of 192 items (64 targets, lures, and foil items each), in which participants engaged in a modified recognition task identifying each item as old, similar, or new items based on the encoding phase. Percentage accuracy is calculated, with higher percentage indicating better function. MST has been used in identifying hippocampal dysfunction associated with aging and dementia and found to be a sensitive and reliable tool in tracking the progression of cognitive decline and treatment efficacy (26, 27).
- Executive function composite (EFC) scales: This combines measures for verbal fluency task, which evaluates language and executive ability for categorical tasks (44); number cancellation task, which assesses visual modality and selective attention (45); and digit symbol substitution task (46) and trail making task (part B) (47), both of which examines visual scanning, executive function, processing, and psychomotor speed. Higher composite scores indicate better executive function. Each neuropsychological test demonstrate good validity and reliability to assess for executive function and track cognitive decline in older adults, for further details see references (48, 49).

#### Secondary Outcome Measures

- Geriatric Depression Scale (GDS) (50): This is a brief 15-item scale, which evaluates depressive symptoms. The GDS score ranges from 0 to 15, with lower scores indicating fewer depressive symptoms. A validation study on the Filipino version of GDS found an adequate internal consistency with Cronbach's α of 0.84 and test re-test reliability ICC of 0.91 (50).
- EuroQoL—Visual Analog Scale (EQVAS) (51): This is used as self-rated scale to assess health-related quality of life. It is a brief, simple, and health-specific measure for quality of life (51, 52); therefore, it is practical for repeated use and appropriate for studies aiming to improve the health of participants. The scores range from 0 to 100, with higher rates indicating better perception of quality of health and life. It reported excellent test–retest reliability ICC of 0.87 and good validity in correlation with multi-item disease-specific QoL measures such as Medical Outcome Scale SF-20 (*r* = 0.70–0.72) and Rotterdam Symptom Check-List (*r* = 0.70) (53).
- Berg Balance Scale (BBS) (54): This consists of 14 tasks evaluating static and functional balance. The score ranges from 0 to 56, with higher scores indicating better function. BBS validation study conducted in older adults with mild to moderate dementia demonstrated excellent validity with Cronbach's α of 0.95 and ICC of 0.99 (55).
- Magnetic resonance imaging (MRI): This will be used to evaluate brain volume and functional connectivity.

### Randomization and Allocation Concealment

Cluster randomization will be used to ensure blinding and avoid contamination of group allocation (33), which was one of the challenges in the quasi-experimental study for INDAK (15). The rationale behind this design is to ensure sociocultural fitness of study to the close-knit and socially collective nature of the Filipino community. Communities are “*barangays*,” the smallest government unit in the Philippines, which is mostly characterized by a high population density (1). People live close to each other, acquainted and frequently interact with one another. They share the same public space and health services (e.g., health center, social center), therefore individual randomization within the same community will limit opportunity of concealing treatment allocation. A barangay is referred to as one cluster in this study.

Randomization will be done after baseline assessment is completed (V1); hence, allocation is concealed to the assessors. Clusters will be randomized to a 1:1 ratio by the study statistician using a computer-generated randomization method, constrained using minimization. That is, each community will be allocated with a probability of 0.8 to the group that minimizes the imbalance at baseline of the means of the primary outcomes. The study statistician will inform the primary investigator of group allocation, and the study coordinator will arrange the delivery of intervention. The outcome assessors will be blinded to group allocation. Their engagement with the participants will be limited only to the day of assessment.

### Multicomponent Intervention

The intervention team consists of physician, nutritionist, nurse, and dance teachers. The components of intervention are:

#### Improving Neurocognition Through Dance and Kinesthetics

Dance teachers are trained professional dance sport specialists with extensive experience in teaching ballroom dancing. INDAK consists of eight ballroom dances organized into 6-week modules with increasing complexity and intensity. The first week of each module introduces basic dance steps or figures then gradually progresses to more complex dance figures. The modules begin first with non-partnered dances allowing familiarization with one's own movement and followed later by dances with a partner, which is more complex and requiring interaction. The dance modules are Reggae, Cha-cha, Samba, Merengue, Bachata, Swing, Tango, and Salsa. The whole dance intervention lasts for 12 months. Each dance session is 60 min comprising of warm-up (5 min), dance proper (45 min), and cool down (10 min). Each session begins with review of previously learned dance figures, then integration of two to three new figures and then rehearsal of all learned dance figures with slow and fast music. The teacher shuffles the figures creating different sequences to test memory and cognitive ability. Concepts such as visualization, musicality exercise, improvisation, and dance recall are used. The nurse will use a logbook to register the frequency and duration of participation of each participant. From a previous study, adverse events were infrequent and limited to musculoskeletal pains in the first 2 weeks of the study (15). To ensure safety, a nurse will always be present at each session and liaise with local hospitals in case of unexpected adverse events. The nurse records vital signs (i.e., blood pressure and heart rate) of all participants before and after the dance. Compliance to INDAK intervention is defined as ≥80% attendance and completion of a full 60-min session for each attendance. Failure to participate or developing physical conditions that limits INDAK participation will be withdrawn from the study.

#### Nutrition Counseling

The dietary advice will be provided by a nutritionist following the Filipino Nutrition Research Institute (FNRI) recommendation called “*Pinggang Pinoy*” or Filipino Plate (56), which is a picture of a 9-inch plate with foods proportionally distributed among the food groups to provide ~1,200–1,500 calories per day. It is advised that half of the plate is composed of green leafy vegetables and one serving of fruit per meal. In general, affordable nutritious food is recommended: high consumption of local fruits and vegetables, brown/purple rice instead of white rice, coconut oil, and other vegetable oils, and fish. General group lectures on healthy diet and individualized counseling for those with identified cardiovascular risks will be done every 3 months. Individualized diet guides developed by FNRI such as high/low caloric, low-sodium, low-cholesterol, and/or diabetes mellitus diet brochures will be provided to the participants depending on their specific needs (available at http://helponline.fnri.dost.gov.ph/help/publications). Diet guides include tips for healthy eating, food selection examples, daily diet plan, and 1-day sample menu. Participants will be asked to use a structured 3-day meal diary for diet monitoring.

#### Vascular Risk Management

After baseline visit (V1), the participants will receive a report on their baseline clinical assessment of vascular risk factors. Participants identified with cardiovascular risks will be examined and advised by study physicians on the management of vascular risks according to standard Filipino clinical guidelines (57–62). When initiation or adjustment of pharmacologic treatment is necessary, participants are given a referral letter to their own physician at the primary health care center for continuity of care. The nurses will monitor participants every month and use a logbook to record their compliance and advise them on a continuing basis. Study physicians and nurses will meet with the participants at the third, sixth, and ninth month to monitor their cardiovascular and metabolic conditions and revise their management plan if necessary. If nurses detect an urgent problem, they will alert participants to see the community physicians.

### Data Processing and Analysis

#### MRI Acquisition

Participants will be scanned in a 3-tesla (3T) MRI scanner (Philips Achieva) using an eight-channel head coil. We will use the Alzheimer's Disease Neuroimaging Initiative (ADNI-3: http://adni.loni.usc.edu/adni-3/) recommended imaging protocols that will allow our data to be comparable with the ADNI centers and good potential for data sharing on open access platforms. A high-resolution 3D T1-weighted sagittal scan/MPRAGE (magnetization prepared gradient echo), and thin T2-weighted and FLAIR (fluid-attenuated inversion recovery) coronal scans are acquired. Functional MRI (task and resting-state) will be collected using a T2^*^ echo-planar imaging (EPI) pulse sequence ADNI-3 Advanced (multiband): TR = 600 milliseconds (ms), TE = 30 ms, flip = 53 degrees, isotropic voxel = 2.5 millimeter (mm), field of view (FOV) = 220 × 220 × 160 mm, 64 slices of simultaneous multi-slice imaging (SMS) = 8 acquired, controlled aliasing in parallel imaging (CAIPI)—shift = 4. Diffusion MRI will be collected using the diffusion tensor imaging (DTI) sequence (multiband): TR = 3,300 ms, TE = 71 ms, isotropic voxel = 2 mm, FOV = 232 × 232 × 160 mm, three shells: b = 500, 1,000, 2,000 seconds/millimeter^2^ (s/mm^2^) (112 total diffusion-weighted directions). The acquisition plane will be rotated 25° with respect to the posterior vertical axis of the brainstem to optimize signal measurements from the cerebellum and neocortices (23). For resting-state fMRI, participants will be asked to keep their eyes open and fixate on a cross (10 min).

**Figure 1:**
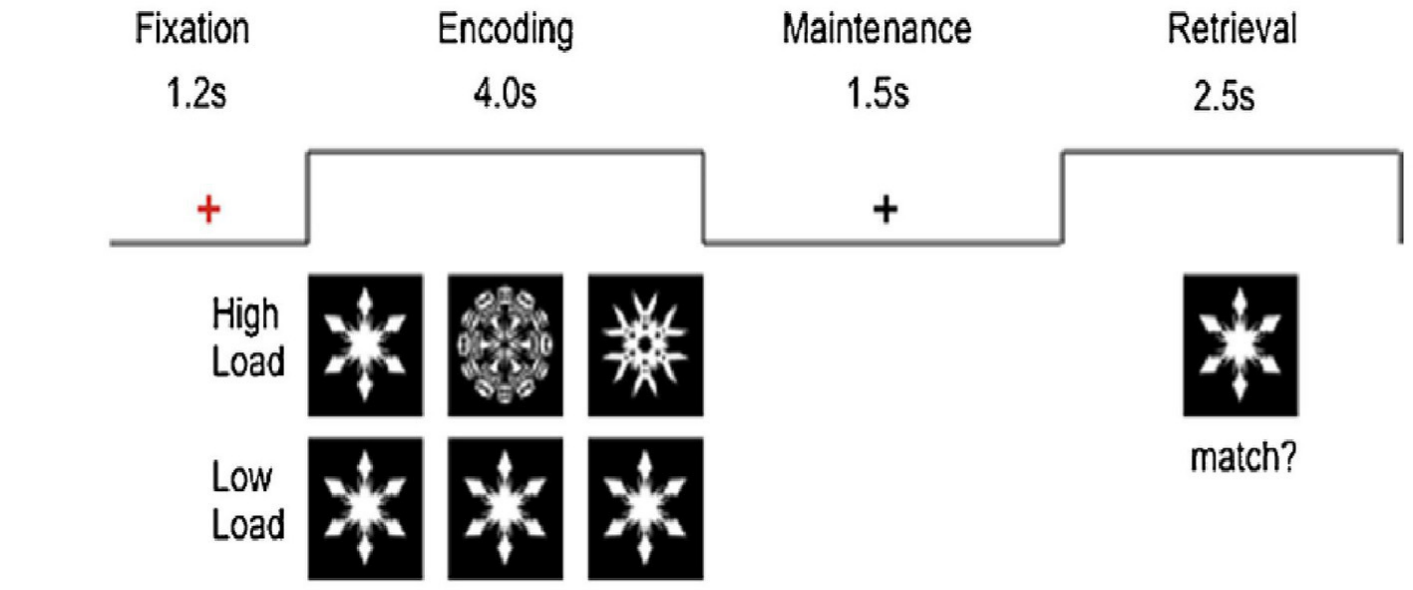
Sternberg visual memory task presented by E-prime in the MRI scanner.

For task fMRI, participants will undergo two runs (6–7 min each) of the visual working memory task (Sternberg paradigm) as described by Ng et al. (24) in the scanner (see Figure 1). There will be alternating blocks of high- or low-load conditions, where they will decide whether the “snow flake” at retrieval matches any of those during encoding with a button press. Each cycle consists of one block of high load and one block of low load that had two trials each. Each block is interleaved by a 1.6-s interval and block duration of 20 s. There will be 10 cycles in each run adding up to a total of 20 high-load and 20 low-load trials. Each run lasts 400 s. E-Prime application (version 2.0; PST) will be used.

#### Magnetic Resonance Imaging Processing and Analysis

Voxel-based morphometry (VBM8) will be applied for overall brain volumetric analysis with the high-resolution MPRAGE T1-weighted image acquired. Tract-Based Spatial Statistics (TBSS) will be applied to diffusion MRI preprocessed with the FMRIB Software Library (FSL) for understanding changes in structural connectivity. The high-resolution MPRAGE scan will also be applied for automated hippocampal volumetric studies using NeuroQuant. The axial thin T2-weighted and FLAIR will be coronal scans that are used for detecting subcortical and deep white mater hyperintensities (WMH). fMRI data will be preprocessed and analyzed using the Statistical Parametric Mapping (SPM-12) software package. All structural and functional images will be reoriented before the EPI data is preprocessed using the conventional procedure in three phases. In phase one, slice timing correction will be corrected to the timing of the middle slice, realignment to the first volume, and coregistration of the structural images to functional images. In phase two of the preprocessing, flow fields are created before the images are smoothed with a Gaussian kernel of 5-mm full width at half maximum (FWHM) and normalized to the Montreal Neurological Institute (MNI) template. Thereafter, first-level analysis (individual statistics) will be computed using the general linear model. This is followed by the second-level analysis (group statistics) in which a random-effect one-sample *t*-test will be applied on all participants in each group to obtain the group activation map. For resting-state data, we will employ a data-driven empirical approach using the group-independent component analysis (GIFT software) to extract the resting-state components, as well as the seed-based approach using functional connectivity software (CONN) toolbox and SPM12 for second-level group analyses. Regions of interest (ROI) analyses will also be conducted. Percent signal change from fMRI maps will be extracted from significant regions to conduct correlational analyses with the cognitive measures (EFC, MST). Similarly, these cognitive measures will also be correlated with the fractional anisotropy (FA) values extracted from the skeletal map from TBSS.

#### Data Management and Statistical Analysis

Data will be encoded independently by two research associates, and inconsistencies will be double checked with the data source to ensure data accuracy. All tests (primary and secondary outcomes) will be analyzed as independent continuous measures; additionally, we will also analyze for executive function composite (EFC) score derived by dividing independent test scores with their maximum possible scores and summing across tests for EFC, as was done in previous studies (63–65).

The study will employ univariate, bivariate, and multivariate statistics. For univariate, descriptive statistics (mean/SD; frequencies/percentages) will be used to summarize sociodemographic and other baseline characteristics. The bivariate analyses that examine underlying relationships between the outcomes and other clinical characteristics are the following: independent *t*-test for continuous variables and Chi square test (or Fisher exact test when ≤ 20 of the cells have the expected count <5) for categorical variables to compare baseline data between the intervention and control groups. Pairwise comparison of the randomization groups per timepoint will also be done to explore differences at different times using independent *t*-test. For multivariate analyses, the study would estimate the effect from baseline to follow-up at 12 months between randomization groups. Since the participants will be assessed three times (at baseline, 6th and 12th months), the study will be using multilevel mixed models to test the significance between group means but controlling for possible confounders (i.e., when age, sex, and education were not successfully controlled for in the randomization design). Necessary diagnostics will also be considered. This multilevel model is appropriate as it can take into account the clustering effect, and the number of clusters in this study is moderately sized (25–30 clusters per arm).

To analyze for the secondary outcomes, RM-ANOVA/RM-ANCOVA will also be used. Point estimates and their corresponding confidence intervals will be evaluated to assess precision and power. Very wide confidence intervals will signify underpowered analyses. Sensitivity analysis will be conducted including imputation of missing data using last observation carried forward or mean imputation. Data management will primarily be the responsibility of the research team. All data will be securely stored and accessible only to the researchers in compliance with the ethics approval of the study.

## Discussion

The FINOMAIN study will carry out the first multicomponent intervention in the Philippines for older adults with MCI. It will add to the limited evidence-based knowledge of complex interventions to reduce dementia risks in LMIC settings. The study aims to provide new scientific evidence to test an innovative model of community-based intervention program evaluating the effect of dance (INDAK) and managing for cardiovascular risks to maintain cognitive performance in a high-risk population. Its conceptualization is based on the rationale that risks for dementia are complex and multifactorial implying that no single approach is effective in addressing overall risks for cognitive decline (66). In this study, the multicomponent intervention is tailored fit to the local needs and cultural settings of the Filipino. Nutrition counseling and cardiovascular risk management are based on local guidelines. Dancing is an enriched way to introduce exercise as a leisure activity and at the same time provides enhanced social and cognitive stimulation (17). It is amenable to the social nature of the Filipino people who love to sing and dance. Furthermore, INDAK is portable, in that no special equipment or facilities are required to conduct it in the communities (15). In a low-resource setting such as the Philippines, this is a low-cost model of intervention that can easily be integrated in the existing services for older adults in the local communities.

This study was designed to target older populations with MCI. The INDAK modules are structured to input cognitively challenging tasks such as visualization, musicality exercise, improvisation, and dance recall as cognitive training elements with increasing intensity and complexity, apart from physical activity. These address limitations of other studies that used dance as intervention in older adults (67). Dance, when compared with repetitive and routine exercise, has superior positive long-term effects on hippocampal plasticity among healthy older adults (18–20). However, brain functional connectivity in response to dance, particularly among those with MCI, remains to be elucidated. Current studies revolving around dance are designed as single-component intervention and mostly explore therapeutic benefits on cognitive performance, and socioemotional and motor functions; however, this study will also explore potential neurological mechanisms of dance and cognition through brain MRI dance, which requires refined motor skills and integration of sensorimotor, proprioception, rhythm, and spatial awareness, which can facilitate cerebro-cerebellar integrity, the largest pathway in the brain. Exploring this connectivity can add understanding to cerebellar functions that explain benefits of dance on various cognitive domains.

## Study Status and Trial Registration

The study started recruitment and randomized eight clusters (72 participants). With the pandemic, the study was stopped and not actively recruiting. It is expected to resume when community restrictions will be eased by the local government. The study was registered with ClinicalTrials.gov on March 10, 2020. As the first participant for the pilot study was enrolled on May 28, 2019, this study was retrospectively registered (https://clinicaltrials.gov/ct2/show/NCT04301544).

## Ethics Statement

The studies involving human participants were reviewed and approved by St. Luke's Medical Center – Institutional Ethics Review Committee. The patients/participants provided their written informed consent to participate in this study.

## Author Contributions

JD, GW, and TP conceptualized the study. JD led the grant application and trial registration and was primarily responsible for the manuscript preparation. MD contributed to the drafting of the manuscript, trial registration, and assisted in the conceptualization. SC conceptualized the methodology and analysis of the brain imaging data of the study. MS advised on the conceptualization of the study and study outcomes. All authors read and approved the last version of the manuscript.

## Funding

This study was supported by grants from the Philippine Institute for Traditional and Alternative Health Care (PITHAC) of the Department of Health. The contributions of SC are partly supported by the Ministry of Education, Singapore, Academic Research Fund (MOE2019-T2-1-019). The funding agencies did not have any role in the study design, nor will they have any role in the implementation or management of the intervention, data collection, analysis and interpretation, manuscript writing, and dissemination of results.

## Conflict of Interest

The authors declare that the research was conducted in the absence of any commercial or financial relationships that could be construed as a potential conflict of interest.

## Publisher's Note

All claims expressed in this article are solely those of the authors and do not necessarily represent those of their affiliated organizations, or those of the publisher, the editors and the reviewers. Any product that may be evaluated in this article, or claim that may be made by its manufacturer, is not guaranteed or endorsed by the publisher.
